# Altersspezifische Trends des risikoreichen Alkoholkonsums in Deutschland: Parallele oder unterschiedliche Verläufe?

**DOI:** 10.1007/s00103-021-03328-7

**Published:** 2021-05-12

**Authors:** Ludwig Kraus, Johanna K. Loy, Nicolas Wilms, Anne Starker

**Affiliations:** 1 IFT Institut für Therapieforschung, Leopoldstr. 175, 80804 München, Deutschland; 2 Department of Public Health Sciences, Centre for Social Research on Alcohol and Drugs, Stockholm University, Stockholm, Schweden; 3 Institute of Psychology, ELTE Eötvös Loránd University, Budapest, Ungarn; 4 Abteilung für Epidemiologie und Gesundheitsmonitoring, Robert Koch-Institut, Berlin, Deutschland

**Keywords:** Riskanter Alkoholkonsum, Rauschtrinken, Trends, Kollektivität, Polarisierung, Risky drinking, Episodic heavy drinking, Trends, Collectivity, Polarisation

## Abstract

**Einleitung:**

Nach der Collectivity-of-Drinking-Cultures-Theorie von Skog finden Veränderungen des Alkoholkonsums in allen Bevölkerungsgruppen und -schichten als parallele Verschiebungen statt. Ziele des vorliegenden Beitrags sind (1) die Darstellung zeitlicher Trends des riskanten Konsums und des episodischen Rauschtrinkens nach Altersgruppen und Geschlecht und (2) die Prüfung, ob die Trends in allen Altersgruppen parallel verlaufen („Kollektivität“) oder zwischen Altersgruppen divergieren („Polarisierung“).

**Methoden:**

Datengrundlage sind 9 Erhebungen des Epidemiologischen Suchtsurveys (ESA) zwischen 1995 und 2018. Als Schwellenwert für riskanten Alkoholkonsum wurde ein täglicher Konsum von mehr als 12 g Reinalkohol bei Frauen beziehungsweise 24 g bei Männern herangezogen. Episodisches Rauschtrinken wurde als Konsum von 5 oder mehr Gläsern Alkohol (ca. 70 g Reinalkohol) an mindestens einem Tag in den letzten 30 Tagen definiert. Lineare Regressionen wurden für die Vorhersage des zeitlichen Effekts auf riskanten Konsum bzw. Rauschkonsum nach Altersgruppen (18–29, 30–39, 40–49 und 50–59 Jahre) und Geschlecht getrennt berechnet und auf Unterschiede geprüft.

**Ergebnisse:**

Die Entwicklungen riskanten Alkoholkonsums nach Altersgruppen verlaufen bei Männern weitgehend parallel, bei Frauen gegenläufig. Die Trends des episodischen Rauschtrinken weisen bei beiden Geschlechtern keine parallele Entwicklung auf: Während in der jüngsten und ältesten Altersgruppe die Prävalenz im Zeitverlauf anstieg, sank sie in den übrigen Altersgruppen.

**Diskussion:**

Vor dem Hintergrund einer generellen Abnahme spricht die Zunahme in den Trends risikoreichen Alkoholkonsums in bestimmten Gruppen für einen Ausbau verhaltenspräventiver Maßnahmen. Zur Fortsetzung der positiven Entwicklung und der Vermeidung einer Trendumkehr sollten zudem auf die Gesamtbevölkerung ausgerichtete Präventionsanstrengungen intensiviert werden, beispielsweise durch Erhöhung der Alkoholsteuer oder Reduktion der Verfügbarkeit von Alkohol.

**Zusatzmaterial online:**

Zusätzliche Informationen sind in der Online-Version dieses Artikels (10.1007/s00103-021-03328-7) enthalten.

## Abstract (en)

**Introduction:**

According to Skog’s collectivity of drinking cultures theory, changes in alcohol consumption in all groups and strata of the population take place as parallel displacement in the distribution of consumption. The aims of the present paper are (1) to illustrate temporal trends in risky drinking and episodic heavy drinking by age and gender and (2) to examine whether the trends are parallel in all age groups (“collectivity”) or diverge between age groups (“polarisation”).

**Methods:**

The data are based on nine surveys of the Epidemiological Survey of Addiction (ESA) between 1995 and 2018. Risky drinking was defined as daily consumption of more than 12 g (for women) or 24 g (for men) of pure alcohol and episodic heavy drinking as consumption of five or more glasses of alcohol (about 70 g pure alcohol) on at least one day in the past 30 days. Linear regressions were calculated separately for age groups (18–29, 30–39, 40–49, and 50–59 years) and gender to predict the temporal effect on risky drinking or episodic heavy drinking and to test trends for differences.

**Results:**

The temporal changes of risky drinking by age group show soft collectivity among men and polarisation among women. Trends in episodic heavy drinking indicate polarisation for both genders; while the prevalence increased in the youngest and oldest age groups, it decreased in all other age groups.

**Discussion:**

In light of a general decrease, the increasing trends in risky drinking in specific groups indicate the need for strengthening behavioural prevention. For the positive development to continue and to avoid a trend reversal, public health measures such as alcohol tax increases and reductions of alcohol availability need to be intensified.

## Einleitung

Hoher Alkoholkonsum steht im Zusammenhang mit einer Vielzahl negativer gesundheitlicher und sozialer Folgen. Laut den Ergebnissen der Global-Burden-of-Disease-(GBD-)Studie ist Alkoholkonsum weltweit einer der Hauptrisikofaktoren für vorzeitige Sterblichkeit und verlorene Lebensjahre infolge von Krankheit und Behinderung [1]. In Westeuropa praktizierte im Jahr 2015 jeder Dritte mindestens einmaliges Rauschtrinken (Konsum von 60 g oder mehr Reinalkohol zu einer Trinkgelegenheit) in den letzten 30 Tagen [2]. Alkoholkonsum ist in Deutschland weitverbreitet und nimmt im europäischen Vergleich gemessen am Pro-Kopf-Verbrauch reinen Alkohols mit 10,9 l pro Jahr einen der Spitzenplätze ein [3]. Im Jahr 2018 berichteten 76,5 % der Männer in den letzten 30 Tagen Alkohol getrunken zu haben, bei Frauen sind es 66,5 %. Riskante Trinkmengen wurden dabei von jeder/jedem 8. berichtet (12,6 %). Die 30-Tage-Prävalenz des episodischen Rauschtrinkens betrug bei Männern 42,8 % und bei Frauen 24,6 % [4]. Trotz der vergleichsweise hohen Prävalenzen des (riskanten) Konsums weisen sowohl der Pro-Kopf-Verbrauch auf der Basis von Verkaufszahlen als auch die Prävalenzwerte aus epidemiologischen Studien in Deutschland auf einen Rückgang des Alkoholkonsums seit 1970 [3] bzw. seit Mitte der 1990er-Jahre hin [5].

Zeitliche Veränderungen des Alkoholkonsums werden in der Regel als Durchschnittswerte ermittelt, entweder über alle Alkoholkonsumentinnen und -konsumenten oder bezogen auf die Gesamtbevölkerung. Sie spiegeln daher nicht notwendigerweise Veränderungen in bestimmten Bevölkerungsgruppen wider und sind somit nicht geeignet, mögliche vulnerable Gruppen zu identifizieren. Nach der Collectivity-of-Drinking-Cultures-Theorie von Skog [6] finden Veränderungen des Alkoholkonsums, gesteuert über soziale Prozesse, in allen Bevölkerungsgruppen und -schichten unisono statt. Dabei kommt es zu einer parallelen Verschiebung der Konsumverteilung in der Bevölkerung, bei der sich das Trinkverhalten aller Konsumgruppen an den jeweiligen Konsummittelwert anpasst (Zu- oder Abnahme). Skog spezifiziert in einem späteren Artikel, dass dabei nicht von einer statischen Situation der gegenseitigen Einflussnahme in und zwischen Gruppen über die Zeit auszugehen ist und dass unterschiedlich starke Veränderungen des Alkoholkonsums zwischen verschiedenen Gruppen nicht auszuschließen sind [7]. Weisen die Trends verschiedener Subgruppen in unterschiedliche Richtungen, spricht man von einer Polarisierung [8, 9], die definiert ist als Gegenteil von parallelen Trends in den verschiedenen Subgruppen. In diesem Fall würde beispielsweise die Prävalenz riskanten Konsums in einer oder mehreren Subgruppen zunehmen, während sie in den anderen abnähme. Subgruppen sind beispielsweise unterschiedliche Statusgruppen oder verschiedene Altersgruppen.

Anhand der zeitlichen Entwicklung von Konsumprävalenzen können Rückschlüsse auf den Umfang aktueller und zukünftig zu erwartender negativer psychischer, physischer und sozialer alkoholbedingter Folgen in der Bevölkerung gezogen werden [10]. Sie sind daher für die Prävention von hoher Bedeutung. Als Indikatoren für die Analyse der Entwicklungen des Alkoholkonsums bieten sich die Schwellenwerte riskanten Konsums aus den Empfehlungen des wissenschaftlichen Kuratoriums der Deutschen Hauptstelle für Suchtfragen (DHS) an [11]. Darüber hinaus wird episodisches Rauschtrinken als weiterer Indikator risikoreichen Konsums in epidemiologischen Untersuchungen weltweit eingesetzt [12]. Anhand von Trinkmengen kann eine Einteilung in schwachen und starken Alkoholkonsum vorgenommen werden [13, 14].

Ziele des vorliegenden Beitrags sind erstens die Darstellung von zeitlichen Trends des riskanten Konsums und des episodischen Rauschtrinkens zwischen 1995 und 2018. Die Entwicklung beider Indikatoren für risikoreichen Alkoholkonsum wird nach Altersgruppen und Geschlecht dargestellt. Auf dieser Basis wird zweitens geprüft, ob diese Trends in allen Altersgruppen parallel verlaufen („Kollektivität“) oder ob sie sich in ihrer Richtung zwischen den Altersgruppen unterscheiden und im Sinne einer „Polarisierung“ divergieren.

## Methoden

### Studiendesign und Stichprobe

Datengrundlage waren 9 Querschnittserhebungen des Epidemiologischen Suchtsurveys (ESA) aus den Jahren 1995, 1997, 2000, 2003, 2006, 2009, 2012, 2015 und 2018. In den Jahren 1995 bis 2003 bildeten deutschsprachige und in Privathaushalten lebende Personen im Alter zwischen 18 und 59 Jahren die Grundgesamtheit des ESA. Mit der Erhebung 2006 wurde die obere Altersgrenze auf 64 Jahre erweitert. Aufgrund der schiefen Altersverteilung in der Bevölkerung, d. h., die Anteile jüngerer sind im Vergleich zu älteren Jahrgängen geringer, wurden die Stichprobengrößen nach Altersgruppen disproportional zum Bevölkerungsanteil gewählt. Die Stichprobenziehung erfolgte 2‑stufig: Zunächst wurden 254 Gemeinden (Sample Points) in ganz Deutschland zufällig ausgewählt, im Anschluss erfolgte eine systematische Zufallsauswahl von Adressen aus den Einwohnermelderegistern. Die Daten wurden in den Jahren 1995–2003 schriftlich und im Jahr 2006 wahlweise zusätzlich telefonisch erhoben. Ab 2009 konnten die Teilnehmenden in einem Mixed-Mode-Design zwischen schriftlichen beziehungsweise webbasierten Fragebögen oder telefonischen Interviews wählen. Die bereinigten Stichprobengrößen variieren zwischen 7822 (1995) und 9267 (2018), die Antwortrate zwischen 65 % (1995) und 41,6 % (2018). Zur Anpassung der Stichprobe an die Verteilung zentraler Merkmale in der Grundgesamtheit wurden die Daten jedes Erhebungsjahres gewichtet. Als Merkmale wurden Alter, Geschlecht, Bundesland und Gemeindegrößenklasse aus dem Mikrozensus für das jeweilige Erhebungsjahr herangezogen (für Details zu den Stichprobenerhebungen siehe [4, 15–22]). Die Änderung der in die Erhebung eingeschlossenen Altersgruppen erlaubt die Analyse zeitlicher Trends für den gesamten Beobachtungszeitraum nur für den Altersbereich von 18 bis 59 Jahren. Trends in der Altersgruppe 60–64 Jahre stehen für die Jahre 2006–2018 zur Verfügung.

### Instrumente

Häufigkeit und Menge des Alkoholkonsums wurden getrennt nach Getränkeart über die letzten 30 Tage erfasst. Als Schwellenwert für riskanten Alkoholkonsum wurde ein täglicher Konsum von mehr als 12 g Reinalkohol bei Frauen und 24 g bei Männern herangezogen [11, 23]. Episodisches Rauschtrinken wurde als Konsum von 5 oder mehr Gläsern Alkohol (entspricht etwa 70 g Reinalkohol) an mindestens einem Tag in den letzten 30 Tagen definiert. Zur Darstellung der Konsumprävalenzen wurden die Schwellenwerte unter Einbeziehung von Personen, die in den letzten 30 Tagen keinen Alkoholkonsum berichteten, berechnet.

### Analysen

Die aus den 9 bevölkerungsrepräsentativen Querschnittserhebungen (1995–2018) geschätzten Prävalenzwerte der Indikatoren des risikoreichen Konsums bilden die Grundlage für die Trendanalysen nach Alter und Geschlecht. In Übereinstimmung mit aktuellen Publikationen zur Überprüfung der Theorie parallel verlaufender Konsumtrends wurde zwischen „starker“ und „schwacher“ Kollektivität unterschieden [8, 9]. In beiden Fällen ist das Vorzeichen der Trends, d. h. die Richtung der zeitlichen Verläufe identisch. Im ersten Fall unterscheiden sich die Trends zwischen den Altersgruppen statistisch nicht signifikant und es wird von einem parallelen Verlauf ausgegangen. Im zweiten Fall zeigen sich Abweichungen von der Parallelität und die Trends zwischen den Altersgruppen unterscheiden sich statistisch signifikant. Bei unterschiedlichen Vorzeichen der Koeffizienten liegen gegenläufige Trends vor und man spricht von einer Polarisierung.

Nach Altersgruppen und Geschlecht getrennt wurden lineare Regressionen für die Vorhersage des zeitlichen Effekts auf riskanten Konsum bzw. Rauschkonsum als abhängige Variable berechnet. Aufgrund der Änderung der in die Studie eingeschlossenen Altersgruppen wurden die Prävalenzen des riskanten Konsums und des Rauschtrinken für die Altersgruppen 18–29, 30–39, 40–49 und 50–59 Jahre über den Zeitraum von 1995 bis 2018 und für die Altersgruppe 60–64 Jahre über den Zeitraum von 2006 bis 2018 berechnet. Personen, die angaben, in den letzten 30 Tagen keinen Alkohol getrunken zu haben, wurden in die Analysen einbezogen, um die Änderungen des Trinkverhaltens der Gesamtbevölkerung in Form von Populationsmittelwerten abzubilden.

Die Trendvergleiche zwischen den Altersgruppen wurden mithilfe von Wald-Tests durchgeführt. Zunächst wurden die Trends der Altersgruppen 18–29, 30–39, 40–49 und 50–59 Jahre über den Zeitraum von 1995 bis 2018 auf Abweichung von 0 getestet und dann gegeneinander auf Unterschiede geprüft (Nullhypothese: kein Unterschied). In einem zweiten Schritt wurde der Trend der Altersgruppe 60–64 Jahre über den Zeitraum von 2006 bis 2018 getestet und dann gegen die Trends der anderen Altersgruppen ebenfalls über den verkürzten Zeitraum von 2006 bis 2018 auf Unterschiede geprüft. Aufgrund multipler Testungen wurde der α‑Wert von 5 % nach Bonferroni korrigiert.

## Ergebnisse

### Trends der Prävalenz riskanten Konsums

**Figure 1:**
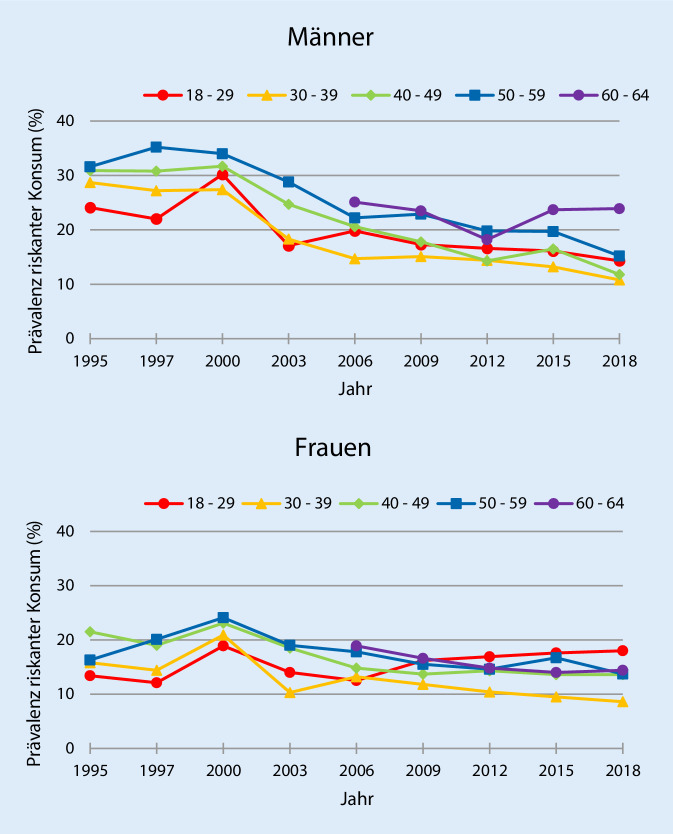

Die Prävalenzwerte des riskanten Konsums mit den dazugehörigen 95 %-Konfidenzintervallen (95 %-KI) nach Altersgruppen, Geschlecht und Surveyjahr (1995–2018) sind im Onlinematerial in Tabelle S1 dargestellt. Im Zeitverlauf 1995 bis 2018 zeigt sich bei beiden Geschlechtern im Mittel eine abnehmende Tendenz des riskanten Alkoholkonsums (Abb. 1). Die Abnahme der Prävalenz riskanten Konsums war bei Männern in der jüngsten und ältesten Altersgruppe geringer als in den anderen Altersgruppen. Bei Frauen nahmen die Prävalenzen in unterschiedlicher Stärke ab. Ausnahme sind die 18- bis 29-Jährigen, bei denen eine Zunahme zu beobachten ist. Bei beiden Geschlechtern sind die Wald-Tests der Unterschiede zwischen allen Altersgruppen statistisch signifikant (Daten nicht dargestellt).

Anhand der Regressionskoeffizienten wird ersichtlich, dass die Prävalenzwerte bei Männern in allen Altersgruppen, wenn auch unterschiedlich stark, abnahmen. Insgesamt weisen die Trends des riskanten Alkoholkonsums im Zeitverlauf bei Männern zwischen den Jahren 1995 bzw. 2006 und 2018 eine schwache Kollektivität auf. Mit dem Anstieg der Prävalenz des riskanten Konsums in der jüngsten Altersgruppe bei gleichzeitig rückläufiger Prävalenz in allen anderen Altersgruppen weisen die Trends des riskanten Alkoholkonsums im Zeitverlauf bei Frauen für die genannten Zeiträume eine Polarisierung auf (Tab. 1).

**Table 1:** 

	Altersgruppen				
	18–29^a^ Betakoeffizient (95 %-KI)	30–39^a^ Betakoeffizient (95 %-KI)	40–49^a^ Betakoeffizient (95 %-KI)	50–59^a^ Betakoeffizient (95 %-KI)	60–64^b^ Betakoeffizient (95 %-KI)
*Riskanter Konsum*					
Männer	−0,469***	−0,823***	−0,971***	−0,879***	−0,059***
	(−0,479; −0,460)	(−0,830; −0,817)	(−0,976; −0,966)	(−0,886; −0,872)	(−0,072; −0,047)
Frauen	0,155***	−0,434***	−0,440***	−0,190***	−0,373***
	(0,150; −0,161)	(−0,440; −0,428)	(−0,445; −0,435)	(−0,199; −0,182)	(−0,379; −0,368)
*Rauschtrinken*					
Männer	0,089***	−0,376***	−0,737***	−0,805***	0,101***
	(0,081; −0,096)	(−0,385; −0,368)	(−0,746; −0,729)	(−0,815; −0,795)	(0,090; 0,112)
Frauen	0,516***	−0,091***	−0,309***	−0,204***	0,108***
	(0,512; 0,520)	(−0,100; −0,083)	(−0,320; −0,299)	(−0,213; −0,194)	(0,101; 0,115)

*** = *p*-Wert <0,001

^a^Zeitraum von 1995 bis 2018

^b^Zeitraum von 2006 bis 2018

### Trends der Prävalenz episodischen Rauschtrinkens

**Figure 2:**
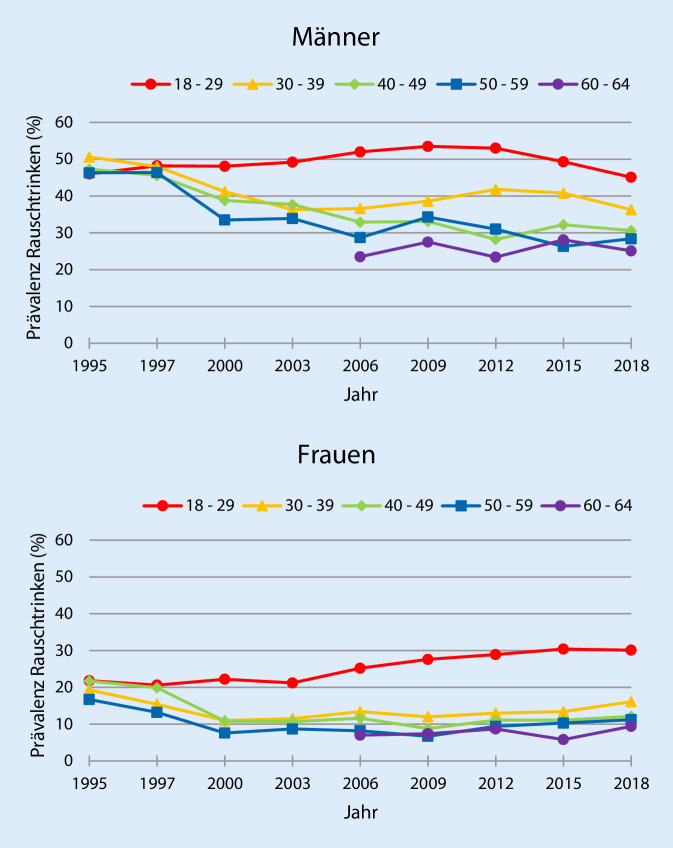

Die Prävalenzwerte des episodischen Rauschtrinkens mit den dazugehörigen 95 %-Konfidenzintervallen nach Altersgruppen, Geschlecht und Surveyjahr (1995–2018) sind im Onlinematerial in Tabelle S2 dargestellt. Bei beiden Geschlechtern zeigt sich im Mittel ein leicht abnehmender Trend des episodischen Rauschtrinkens, wenn auch auf unterschiedlichem Niveau (Abb. 2). Die Abnahme fiel bei den Männern je nach den Altersgruppen mit −4 bis −18 Prozentpunkten etwas höher aus als bei den Frauen (−3 bis −15 Prozentpunkte). Die Prävalenzwerte des episodischen Rauschtrinkens der 60- bis 64-Jährigen waren zwischen den Jahren 2006 und 2018 bei beiden Geschlechtern am niedrigsten und sind zwischen den Jahren 1995 und 2018 bei den 18- bis 29-Jährigen am höchsten, wobei vor allem bei den Frauen eine Zunahme zu beobachten ist. Das spiegelt sich auch anhand der Regressionskoeffizienten wider. Diese weisen darüber hinaus auch für die 60- bis 64-Jährigen positive Werte und damit eine steigende Tendenz auf (Tab. 1). Bei beiden Geschlechtern finden sich zwischen allen Altersgruppen statistisch signifikante Abweichungen in den Trends (Daten nicht dargestellt). In Verbindung mit Tab. 1 wird deutlich, dass die Trends der 30- bis 39-Jährigen, der 40- bis 49-Jährigen sowie der 50- bis 59-Jährigen bei beiden Geschlechtern einen abnehmenden Trend aufweisen, wenn auch unterschiedlich stark. Insgesamt liegt bei beiden Geschlechtern zwischen den Jahren 1995 und 2018 eine Polarisierung im zeitlichen Verlauf des Rauschtrinkens vor.

## Diskussion

Im vorliegenden Beitrag wurden die Trends der Indikatoren risikoreichen Alkoholkonsums unter Anwendung linearer Regressionsmodelle getrennt nach Altersgruppen und Geschlecht verglichen. Im Zeitverlauf stieg die Prävalenz des riskanten Konsums für die jüngste Altersgruppe bei den Frauen an. Für alle anderen Altersgruppen beider Geschlechter nahm sie zwischen den Jahren 1995 bzw. 2006 und 2018 ab, die jüngste und älteste Altersgruppe wiesen dabei die geringsten Reduktionen auf. Demnach liegt in der Entwicklung der Prävalenz riskanten Alkoholkonsums bei Männern schwache Kollektivität und bei Frauen Polarisierung vor. Für denselben Zeitraum zeigt sich bezüglich des episodischen Rauschtrinkens bei beiden Geschlechtern ein polarisierender Verlauf: Während in der jüngsten und ältesten Altersgruppe die Prävalenz anstieg, sank sie in den übrigen Altersgruppen.

### Einordnung in bisherige Studienlage

Eine Besonderheit der vorliegenden Studie ist die Betrachtung verschiedener Altersgruppen in Hinblick auf die Kollektivität unter Verwendung der Prävalenz riskanter Trinkmengen und episodischen Rauschtrinkens als Indikatoren risikoreichen Alkoholkonsums. In bisherigen Analysen zu Kollektivität versus Polarisierung wurden in der Regel die Veränderungen der Trinkmenge über verschiedene Konsumniveaus (leichter bis starker Konsum) einbezogen. Rossow und Kollegen [14] fanden in ihrer Studie kollektive Veränderungen der Trinkmengen in Finnland (1968 bis 2008), Norwegen (1973 bis 2004) und den USA (1979 bis 2010), d. h., in allen 3 Ländern wiesen die zeitlichen Trends des Durchschnittskonsums von Gruppen mit leichtem und starkem Konsum keine Unterschiede in Richtung und Stärke auf. Diese Beobachtung galt unabhängig davon, ob der Pro-Kopf-Konsum des Landes sank oder stieg. Im Gegensatz dazu berichteten Holmes und Kollegen [9] für das Vereinigte Königreich eine Mischung aus Kollektivität und Polarisierung in den Trends zwischen den Jahren 1984 und 2011 über Gruppen mit unterschiedlichen Trinkmengen. Die Ergebnisse ihrer Analysen nach Alter, Periode und Kohorte zeigten Unterschiede in den Trends von Personen mit leichtem und starkem Konsum hinsichtlich des zeitlichen Verlaufs (Periode) und nach Kohorte, d. h., die durchschnittliche Trinkmenge stieg in einigen Altersgruppen, während sie in anderen fiel.

Weitere Studien zur Kollektivität des Trinkverhaltens konzentrierten sich bislang vorwiegend auf Jugendliche: So wurden in verschiedenen Untersuchungen Hinweise auf kollektives Trinkverhalten Jugendlicher in Schweden und Norwegen gefunden [14, 24–27]. Im Vereinigten Königreich weisen aktuelle Ergebnisse auf eine lediglich schwache Kollektivität hin [8] und Hallgren und Kollegen [28] fanden unter Verwendung von Daten einer Schülerbefragung in Stockholm polarisierende Trends. Im internationalen Vergleich ergibt sich somit bei Jugendlichen insgesamt ein gemischtes Bild hinsichtlich der Veränderung der Trinkmenge in Gruppen Jugendlicher mit starkem und schwachen Konsum.

### Bedeutung für die Alkoholpolitik

Die unterschiedlichen Trends bzw. gegenläufigen Entwicklungen über Altersgruppen verdeutlichen, dass in Bezug auf Maßnahmen zur Verringerung des riskanten Alkoholkonsums spezifischer Handlungsbedarf für einzelne Altersgruppen besteht. Auch die divergierenden Trends der Prävalenz episodischen Rauschtrinkens deuten in diese Richtung, vor allem, wenn man davon ausgeht, dass sich Trinkmuster im Jugend- und jungen Erwachsenenalter ausbilden und im weiteren Lebensverlauf beibehalten werden [29].

Die Ergebnisse schwacher Kollektivität oder Polarisierung weisen darauf hin, dass Bedarf für präventive Maßnahmen besteht, die sich speziellen Risikogruppen, wie etwa Personen mit starkem Konsum, widmen [8, 30]. Dies soll verhindern, dass der risikoreiche Konsum in bestimmten Subgruppen der Bevölkerung steigt oder auf hohem Niveau stagniert und alkoholbedingte Schäden möglicherweise auch bei einer Reduktion des Gesamtkonsums nicht zurückgehen. Subgruppenanalysen des Trinkverhaltens liefern somit Hinweise für die Erklärung gegenläufiger Trends von Alkoholkonsum und alkoholbedingten Schäden [31, 32].

Nationale Unterschiede zu parallelen versus gegenläufige Entwicklungen des Trinkverhaltens in Subgruppen müssen vor dem Hintergrund nationaler Unterschiede im Trend des Gesamtkonsums und in den Präventionsstrategien diskutiert werden. Die in der Studie von Rossow und Kollegen [14] vorgenommenen Ländervergleiche zwischen Norwegen, Finnland und den USA legen nahe, dass die Ergebnisse kollektiver Änderungen des Trinkverhaltens nicht davon beeinflusst werden, ob der Gesamtkonsum in einem Land einen zu- oder abnehmenden Trend aufweist. Die Alkoholpolitik nordeuropäischer Länder setzt jedoch vorwiegend auf Bevölkerungsebene an, d. h., es wird eine Reduktion der Gesamtmenge getrunkenen Alkohols (Pro-Kopf-Konsum) angestrebt. In diese Tradition kann man weitgehend auch die USA einordnen, die historisch auf den starken Einfluss der Temperenz-Bewegung (soziale Bewegung gegen den Genuss alkoholischer Getränke) zurückgeht [33]. Public-Health-Strategien zur Reduktion des Alkoholkonsums, wie z. B. hohe Preispolitik oder Verfügbarkeitsbeschränkungen, haben sich als wirksame Instrumente zur Verringerung sowohl des Alkoholkonsums als auch der damit verbundenen Schäden erwiesen [34]. Die Ergebnisse kollektiver Veränderungen des Alkoholkonsums unabhängig von der Trinkmenge könnten daher auch als die Folge von Public-Health-Strategien interpretiert werden, da diese auf alle Konsumentengruppen gleichermaßen wirken. Die Befunde zu divergierenden Konsumtrends in unterschiedlichen Subgruppen [13, 35, 36] in Ländern, die sich eher durch eine liberale, wenig einschränkende Alkoholpolitik auszeichnen (zu Deutschland siehe [37]), deuten in diese Richtung.

Die Abweichungen der von Skogs Theorie vorausgesagten kollektiven Änderung des Trinkverhaltens, auch in nordeuropäischen Ländern mit einer strikten Alkoholpolitik [13, 28, 35, 36, 38], machen deutlich, dass der Public-Health-Ansatz auf Bevölkerungsebene (Verhältnisprävention) nicht ausreicht. Stattdessen sind zusätzlich verhaltenspräventive Maßnahmen nötig, die spezifische Gruppen, die von den kollektiven Veränderungen abweichen, wie etwa Hochkonsumgruppen, im Blick haben [30, 39].

### Limitationen

Bei der hier vorgenommenen Prüfung der Hypothese einheitlicher Änderungen des Trinkverhaltens in Subgruppen ist zu beachten, dass sie in der aktuellen Analyse sowie in allen uns bekannten Studien auf der Grundlage wiederholter Messungen (Surveys) mit unabhängigen Stichproben getestet wurde und die Ergebnisse somit weder individuelle Änderung im Trinkverhalten noch Änderungen im Abstinenzverhalten berücksichtigen [10]. Einschränkungen der Validität der Ergebnisse ergeben sich weiterhin aus dem Umstand, dass die verwendeten Daten zu Häufigkeit und Menge konsumierten Alkohols auf Selbstangaben basieren. Dadurch kann es zu Verzerrungen u. a. aufgrund von Erinnerungslücken, der fehlerhaften Einschätzung von Gläsergrößen und sozial erwünschtem Antwortverhalten kommen. Da davon auszugehen ist, dass sich diese Einflüsse über die Zeit nicht wesentlich verändert haben und sich gleichermaßen auf alle Erhebungen auswirken, können die beobachteten Trends als unabhängig von diesen Einflüssen angesehen werden, auch wenn mögliche Verzerrungen insbesondere in Form einer Unterschätzung der Prävalenzwerte nicht auszuschließen sind. Antwortraten von etwa 50 % gelten im internationalen Vergleich als durchaus akzeptabel, dennoch bergen sie das Risiko systematischer Verzerrungen. Gleiches trifft für die Anwendung unterschiedlicher Befragungsmodi innerhalb bzw. zwischen den Erhebungen (schriftlich, telefonisch, webbasiert) zu. Die Nichtteilnahme an den ESA-Befragungen schlägt sich in erster Linie in einer überdurchschnittlich hohen Beteiligung von Personen aus den mittleren Bildungs- und Einkommensschichten nieder, was die Repräsentativität der Studie für die proportional weniger häufig vertretenen geringen und hohen Bildungs- und Einkommensschichten verringert („Mittelschichtsbias“). Da sich der Mittelschichtsbias über die Jahre kaum verändert hat, ist auch hier davon auszugehen, dass die Trendergebnisse davon unbeeinflusst sind. Einflüsse durch Änderungen in den Erhebungsmodalitäten sind nicht vollständig auszuschließen, Sensitivitätsanalysen weisen jedoch auf nur geringe Unterschiede im Antwortverhalten hin [4]. Die Verwendung der 30-Tage-Konsumprävalenz birgt zudem die Gefahr geringer Validität, etwa aufgrund von Unterschätzung sowie jahreszeitlichen Schwankungen. Auch hier sind die Auswirkungen in Hinblick auf die Trends als eher gering einzustufen. Die Erhebungen des ESA erstreckten sich über mehrere Monate und fanden im jeweils gleichen Jahreszeitraum von März bis August statt.

## Schlussfolgerung

Der vorliegende Beitrag liefert Erkenntnisse zur Entwicklung risikoreichen Konsums in verschiedenen Altersgruppen in Deutschland. Einem generell abnehmenden Trend risikoreichen Konsumverhaltens steht eine Zunahme des Anteils von Personen mit einem risikoreichen Konsumverhalten in bestimmten Gruppen entgegen. Dies spricht für einen Ausbau verhaltenspräventiver Maßnahmen. Trotz der positiven Entwicklung ist Deutschland ein Hochkonsumland mit einer vergleichsweise liberalen Alkoholpolitik [37]. Zur Fortsetzung der positiven Entwicklung und zur Vermeidung einer Trendumkehr sollten zudem auf die Gesamtbevölkerung ausgerichtete Präventionsanstrengungen intensiviert werden, beispielsweise durch Steuererhöhung oder Reduktion der Verfügbarkeit von Alkohol.

## Supplementary Information


