# Supplementary material for: Age-specific trends in risky drinking in Germany: collectivity or polarisation?
Source: Bundesgesundheitsblatt Gesundheitsforschung Gesundheitsschutz. 2021 May 12;64(6):652–9. [Article in German] doi: 10.1007/s00103-021-03328-7 (PMC8187186; doi:10.1007/s00103-021-03328-7)
Supplement: Supplementary file 1 [file 103_2021_3328_MOESM1_ESM.pdf]

Bundesgesundheitsblatt 6/2021

Elektronisches Zusatzmaterial zum Beitrag:

Altersspezifische Trends des risikoreichen Alkoholkonsums in Deutschland:

Parallele oder unterschiedliche Verläufe?

Ludwig Kraus[1,2,3], Johanna K. Loy[1], Nicolas Wilms[1], Anne Starker[4]

[1] IFT Institut für Therapieforschung, München, Deutschland
[2] Department of Public Health Sciences, Centre for Social Research on Alcohol and Drugs, Stockholm University, Stockholm, Schweden
[3] Institute of Psychology, ELTE Eötvös Loránd University, Budapest, Ungarn
[4] Abteilung für Epidemiologie und Gesundheitsmonitoring, Robert Koch-Institut, Berlin, Deutschland

Korrespondenzadresse
Prof. Dr. phil. Ludwig Kraus
IFT Institut für Therapieforschung
Leopoldstr. 175
80804 München
Deutschland
kraus@ift.de

Inhalt:

Tab. S1: Schätzung der 30-Tage-Prävalenz des riskanten Alkoholkonsums in der Bevölkerung im Zeitraum von 1995 bis 2018 nach Altersgruppen und Geschlecht
Tab. S2: Schätzung der 30-Tage-Prävalenz des episodischen Rauschtrinkens von Alkohol in der Bevölkerung im Zeitraum von 1995 bis 2018 nach Altersgruppen und Geschlecht

Tab. S1: Schätzung der 30-Tage-Prävalenz des riskanten Alkoholkonsums in der Bevölkerung im Zeitraum von 1995 bis 2018 nach Altersgruppen und Geschlecht. Datenquelle: Epidemiologischer Suchtsurvey (ESA)

**Riskanter Konsum**																											
**Männer**	**1995**			**1997**			**2000**			**2003**			**2006**			**2009**			**2012**			**2015**			**2018**		
**Alter**	**n**	**%**	**95 % KI**	**n**	**%**	**95 % KI**	**n**	**%**	**95 % KI**	**n**	**%**	**95 % KI**	**n**	**%**	**95 % KI**	**n**	**%**	**95 % KI**	**n**	**%**	**95 % KI**	**n**	**%**	**95 % KI**	**n**	**%**	**95 % KI**
**18 - 29**	194	24,1	[20.8; 27.7]	201	22,0	[18.3; 26.2]	202	30,2	[26.7; 33.9]	223	17,1	[15.1; 19.4]	248	19,8	[17.4; 22.4]	245	17,3	[15.5; 19.3]	221	16,6	[14.5; 18.9]	261	16,1	[14.2; 18.2]	228	14,3	[12.6; 16.3]
**30 - 39**	271	28,7	[25.6; 32.0]	295	27,2	[23.8; 30.8]	249	27,4	[24.6; 30.4]	119	18,3	[15.3; 21.7]	78	14,7	[11.7; 18.2]	96	15,1	[12.5; 18.2]	72	14,4	[11.3; 18.2]	81	13,2	[10.9; 16.0]	79	10,8	[8.5; 13.7]
**40 - 49**	271	30,9	[27.2; 34.8]	254	30,8	[26.6; 35.4]	273	31,7	[28.4; 35.2]	187	24,7	[21.4; 28.4]	127	20,6	[17.4; 24.3]	110	17,8	[14.9; 21.1]	102	14,3	[11.8; 17.2]	108	16,5	[13.7; 19.7]	78	11,8	[9.5; 14.6]
**50 - 59**	317	31,6	[28.4; 35.0]	355	35,2	[31.0; 39.6]	318	34,0	[30.7; 37.5]	217	28,8	[25.5; 32.4]	145	22,2	[19.2; 25.6]	125	22,9	[19.6; 26.5]	159	19,8	[16.9; 23.0]	139	19,7	[16.7; 23.1]	108	15,2	[12.7; 18.0]
**60 - 64**	-	-	-	-	-	-	-	-	-	-	-	-	105	25,1	[21.1; 29.4]	74	23,5	[19.3; 28.3]	88	18,2	[14.9; 22.2]	95	23,7	[19.9; 28.1]	105	23,9	[20.3; 28.0]
**Frauen**	**1995**			**1997**			**2000**			**2003**			**2006**			**2009**			**2012**			**2015**			**2018**		
**Alter**	**n**	**%**	**95 % KI**	**n**	**%**	**95 % KI**	**n**	**%**	**95 % KI**	**n**	**%**	**95 % KI**	**n**	**%**	**95 % KI**	**n**	**%**	**95 % KI**	**n**	**%**	**95 % KI**	**n**	**%**	**95 % KI**	**n**	**%**	**95 % KI**
**18 - 29**	144	13,4	[11.1; 16.3]	123	12,1	[9.5; 15.4]	177	18,9	[16.6; 21.6]	232	14,0	[12.1; 16.0]	200	12,5	[10.8; 14.3]	282	16,2	[14.5; 18.2]	281	16,9	[15.1; 18.9]	307	17,6	[15.7; 19.8]	333	18,0	[16.1; 20.0]
**30 - 39**	223	15,8	[13.6; 18.3]	218	14,4	[12.0; 17.1]	247	20,9	[18.3; 23.7]	91	10,3	[8.5; 12.4]	94	13,2	[10.8; 16.0]	98	11,8	[9.8; 14.2]	87	10,4	[8.4; 12.9]	80	9,5	[7.6; 11.7]	74	8,6	[6.9; 10.7]
**40 - 49**	229	21,5	[18.7; 24.5]	197	19,0	[15.7; 22.9]	234	23,1	[20.5; 26.0]	156	18,5	[16.1; 21.3]	119	14,8	[12.4; 17.4]	102	13,7	[11.4; 16.3]	126	14,3	[12.0; 16.9]	124	13,6	[11.4; 16.2]	117	13,6	[11.5; 15.9]
**50 - 59**	161	16,3	[13.9; 19.0]	172	20,1	[16.6; 24.2]	230	24,1	[21.7; 26.8]	150	19,0	[16.3; 22.0]	138	17,8	[15.1; 20.8]	103	15,5	[12.9; 18.4]	151	14,6	[12.5; 16.9]	146	16,7	[14.3; 19.4]	123	13,7	[11.6; 16.0]
**60 - 64**	-	-	-	-	-	-	-	-	-	-	-	-	81	18,9	[15.2; 23.3]	60	16,6	[13.1; 20.8]	84	14,8	[12.0; 18.1]	75	14,0	[11.1; 17.6]	71	14,4	[11.5; 17.8]

n Anzahl; ***KI*** Konfidenzintervall

Tab. S2: Schätzung der 30-Tage-Prävalenz des episodischen Rauschtrinkens von Alkohol in der Bevölkerung im Zeitraum von 1995 bis 2018 nach Altersgruppen und Geschlecht. Datenquelle: Epidemiologischer Suchtsurvey (ESA)

Episodisches Rauschtrinken																											
**Männer**	**1995**			**1997**			**2000**			**2003**			**2006**			**2009**			**2012**			**2015**			**2018**		
**Alter**	**n**	**%**	**95 % KI**	**n**	**%**	**95 % KI**	**n**	**%**	**95 % KI**	**n**	**%**	**95 % KI**	**n**	**%**	**95 % KI**	**n**	**%**	**95 % KI**	**n**	**%**	**95 % KI**	**n**	**%**	**95 % KI**	**n**	**%**	**95 % KI**
**18 - 29**	383	46,0	[42.1; 49.9]	402	48,2	[43.5; 53.0]	346	48,1	[44.3; 52.0]	657	49,2	[46.1; 52.4]	652	52,0	[48.8; 55.2]	757	53,5	[50.8; 56.2]	709	53,0	[50.0; 55.9]	826	49,3	[46.7; 52.0]	724	45,1	[42.6; 47.8]
**30 - 39**	457	50,6	[47.0; 54.2]	483	48,0	[43.8; 52.2]	404	41,2	[37.2; 45.2]	241	36,3	[32.7; 40.1]	194	36,6	[32.5; 40.9]	247	38,6	[34.5; 42.9]	215	41,8	[37.4; 46.3]	251	40,8	[37.0; 44.6]	265	36,3	[32.8; 40.0]
**40 - 49**	393	47,3	[43.3; 51.3]	366	45,6	[40.6; 50.6]	358	38,8	[35.0; 42.7]	291	37,7	[34.0; 41.5]	199	32,9	[29.1; 36.8]	205	33,1	[29.3; 37.2]	203	28,2	[25.1; 31.5]	217	32,2	[28.7; 36.0]	212	30,6	[27.0; 34.4]
**50 - 59**	458	46,3	[42.6; 50.0]	420	46,4	[41.8; 51.0]	339	33,5	[30.0; 37.2]	255	33,9	[30.7; 37.2]	190	28,7	[25.0; 32.7]	190	34,3	[30.4; 38.5]	252	31,0	[27.6; 34.6]	195	26,3	[23.0; 29.9]	204	28,4	[25.1; 31.9]
**60 - 64**	-	-	-	-	-	-	-	-	-	-	-	-	98	23,5	[19.4; 28.1]	88	27,5	[22.7; 32.9]	116	23,4	[19.7; 27.5]	115	28,1	[24.2; 32.4]	110	25,1	[21.3; 29.4]
**Frauen**	**1995**			**1997**			**2000**			**2003**			**2006**			**2009**			**2012**			**2015**			**2018**		
**Alter**	**n**	**%**	**95 % KI**	**n**	**%**	**95 % KI**	**n**	**%**	**95 % KI**	**n**	**%**	**95 % KI**	**n**	**%**	**95 % KI**	**n**	**%**	**95 % KI**	**n**	**%**	**95 % KI**	**n**	**%**	**95 % KI**	**n**	**%**	**95 % KI**
**18 - 29**	215	21,8	[18.8; 25.2]	190	20,6	[17.1; 24.6]	220	22,2	[19.5; 25.1]	378	21,2	[18.8; 23.7]	429	25,2	[22.8; 27.8]	495	27,6	[25.4; 30.0]	505	28,9	[26.5; 31.5]	566	30,4	[28.0; 32.8]	579	30,1	[27.8; 32.5]
**30 - 39**	251	19,3	[16.9; 22.1]	206	15,4	[12.9; 18.2]	138	11,0	[9.3; 12.9]	104	11,5	[9.6; 13.8]	97	13,4	[10.8; 16.3]	102	12,0	[10.0; 14.3]	112	13,0	[10.9; 15.4]	116	13,4	[11.4; 15.8]	144	16,1	[13.8; 18.7]
**40 - 49**	224	21,7	[18.9; 24.8]	174	19,9	[16.3; 24.1]	118	10,8	[9.0; 13.0]	89	10,7	[8.6; 13.2]	93	11,6	[9.4; 14.3]	67	8,8	[7.0; 11.1]	100	11,1	[9.1; 13.4]	105	11,1	[9.2; 13.4]	106	12,1	[10.2; 14.4]
**50 - 59**	176	16,7	[14.3; 19.4]	117	13,2	[10.4; 16.7]	77	7,6	[5.8; 9.7]	70	8,7	[6.9; 11.0]	64	8,2	[6.4; 10.5]	45	6,7	[5.0; 9.0]	98	9,4	[7.7; 11.3]	91	10,3	[8.4; 12.5]	101	11,2	[9.3; 13.4]
**60 - 64**	-	-	-	-	-	-	-	-	-	-	-	-	28	7,0	[4.9; 10.0]	28	7,4	[5.2; 10.3]	50	8,7	[6.7; 11.1]	31	5,8	[4.0; 8.3]	46	9,4	[6.9; 12.5]

n Anzahl; ***KI*** Konfidenzintervall
